# Editorial: Advances in peanut research

**DOI:** 10.3389/fpls.2023.1180009

**Published:** 2023-04-04

**Authors:** Tingting Chen, Guowei Li, Hong Zhang

**Affiliations:** ^1^ Guangdong Key Laboratory of Plant Molecular Breeding, South China Agricultural University, Guangzhou, China; ^2^ Institute of Crop Germplasm Resources, Shandong Academy of Agricultural Sciences, Jinan, China; ^3^ Department of Biological Sciences, Texas Tech University, Lubbock, TX, United States

**Keywords:** peanut, germplasm, phenotyping, QTLs/genes mapping, functional analysis

Peanut (*Arachis hypogaea* L.) is an important oilseed crop, which is cultivated in over 100 countries. High yield, nutritional quality, resistance to biotic and abiotic stresses, and mechanization in cultivation and harvest are still the goals of peanut breeding in many countries. Peanut is an allotetraploid legume (AABB, 2n = 4x = 40, 2.7 Gb) that derived from hybridization between two diploid peanuts *A. duranensis* (AA) and *A. ipaensis* (BB) in the region from southern Bolivia to northern Argentina about 9400 years ago (Bertioli et al., 2019). With rapid development of bioinformatic tools, sequencing of several peanut genomes, including three cultivated allotetraploid peanuts (i.e., Tiffrunner, Shitouqi and Fuhuasheng) and two ancestral diploid species, made detailed studies of Functional genomics in peanuts possible (Bertioli et al., 2016; Bertioli et al., 2019; Chen et al., 2019; Zhuang et al., 2019). Analyzing the genetic basis and developing molecular markers of important traits will likely provide the essential knowledge for genetic improvement of peanuts. In order to speed up the genetic improvement of peanut, the Research Topic of “Advances in Peanut Research” aimed at addressing gaps in the research areas of genetics, Functional genomics, and germplasm development in the *Arachis* genus, which will lead to a better understanding of the yield, seed quality, response to abiotic and biotic stresses of peanuts, thereby to achieve sustainable production of peanut in the future.

In this collection, several works studied regulatory network of yield, abiotic and biotic stress related traits and molecular markers of seed quality. Peanut yield is one of the most important agronomic traits, which is affected by the total pod number per plant, 100-pod weight, and shelling percentage. Wu et al. conducted a comprehensive transcriptome analysis at five different stages of pod development in YH15, W1202 and their RIL lines S181523 and S181517 with significantly different pod sizes. They showed that the regulatory network of pod development is composed of transcription factors, hormones and the mitogen-activated protein kinase (MAPK) signaling pathways. Peanut blooms above the ground but bears fruits underground, which is the necessary condition of both darkness and mechanical stress after its peg penetrates the ground. Cui et al. studied the changes of gene expression during the reverse process of peg penetration and aerial pods without mechanical pressure and darkness, which revealed that differentially expressed genes (DEGs) were significantly enriched in photosynthesis, photosynthesis-antenna proteins, plant-pathogen interaction, DNA replication, and circadian rhythm pathways. Importantly, they proposed that two signal transduction pathways are possibly involved in peanut geocarpy, namely, one begins in chloroplast with signaling through phosphorylation cascade, and the other initiated in response to abiotic stresses that involves calcium signaling, phosphorylation, and ubiquitination. High oleic acid content, as a measure of peanut seed quality, could extend shelf life of peanut oil and benefit human health, which was regulated by *ahFAD2A* and *ahFAD2B* mutant alleles. Kamdar et al. introgressed mutant alleles *ahFAD2A* and *ahFAD2B* from SunOleic 95R into two popular peanut cultivars, GG-7 and TKG19A by markers-assisted selection (MAS) and backcrossing (MABC). They developed 22 lines with increased oleic acid and unchanged mineral and vitamin composition except potassium content. Moreover, pooled pod yield of two introgression lines had over 10% higher than control varieties. Many fungal diseases cause significant loss in peanut yield and reduce the quality of peanuts, especially *Aspergillus flavus*. In order to study the resistance mechanism to *Aspergillus flavus* in peanut, Cui et al. found that pathogenesis-related proteins, serine/threonine kinase, MAPK kinase, pattern recognition receptors play important roles in peanut resistance to *A. flavus* via comparative transcriptome analysis and weighted gene co-expression network analysis (WGCNA). The yield and quality of peanut are often restricted by certain soil factors, especially nitrogen (N) deficiency and soil compaction (Yang et al.). They revealed that genes and metabolites in amino acid metabolism, TCA cycle, lipids metabolism, and isoflavonoid biosynthesis pathway are involved in response to soil compaction and N deficiency through transcriptome and metabolome analyses in peanut root cells. MADS-box transcription factors are reported to be widely involved in regulating plant growth and development and tolerance to abiotic stresses. MIKC-type MADS-box genes family was identified in both cultivated and wild-type peanut genomes (Mou et al.). Expression pattern analysis showed that *AhMADS* genes are involved in different abiotic stresses via hormonal signaling.

In summary, studies in this Research Topic collection reported research on diverse traits, including pod development, seed quality, abiotic and biotic stress responses, which are comprehensive and provide important knowledge to peanut researchers. Nevertheless, our understanding of the genetic bases for many important agronomic traits in peanut is still very limited, and many outstanding problems still remain. For example, artificial selection during intensive breeding has dramatically enhanced the yield, yet resulted in a narrow genetic base for peanut breeding. Landraces and wild species of *Arachis*, as a strategic source of diversity, may benefit food security, climate change adaptation and sustainability. Therefore, utilization of landraces and wild species in *Arachis* should be paid more attention. Combined multi-omics analysis should be widely applied in the mining of alleles that could contribute to novel agronomic trait improvement. With the rapid development of the omics technologies, analyzing omics data to clarify molecular regulatory mechanisms of complex traits is becoming possible. Moreover, robust phenotyping is essential because it is the main basis for utilization of germplasm resources. Ultimately, further understanding of peanut gene function and elucidating the molecular mechanism of agronomic trait development will provide the theoretical basis for improving the yield and quality of peanuts.

## Author contributions

All authors listed have made a substantial, direct, and intellectual contribution to the work and approved it for publication.
